# Drug-induced male infertility: a real-world study using FAERS and EudraVigilance databases

**DOI:** 10.3389/fphar.2026.1765071

**Published:** 2026-01-29

**Authors:** Zhuozhi Gong, Jing He, Qiujian Feng, Dong Liu, Shengjing Liu

**Affiliations:** 1 Department of Andrology, Xiyuan Hospital of China Academy of Chinese Medical Sciences, Beijing, China; 2 School of Traditional Chinese Medicine, Southern Medical University, Guangzhou, China; 3 China Institute for History of Medicine and Medical Literature, China Academy of Chinese Medical Sciences, Beijing, China

**Keywords:** EudraVigilance, FAERS, male infertility, pharmacovigilance, reproductive toxicity

## Abstract

**Objective:**

To systematically identify medications potentially causing male infertility or sperm abnormalities and provide risk alerts for clinical practice.

**Methods:**

A pharmacovigilance study was conducted using the FAERS database (Q1 2004–Q2 2025) and EudraVigilance (EV) database (January 2002–October 2025). Adverse events related to male reproductive toxicity were screened using the MedDRA dictionary. Drug safety signals were detected using ROR, PRR, IC, and EBGM, with reliability enhanced through cross-database validation and dechallenge/rechallenge analyses.

**Results:**

The study included 1,955 FAERS cases and 1,384 from EV, with a median patient age of 35 years and 37% being reproductive-age males (18–44 years). The median time to event onset was 132 days, consistent with the spermatogenic cycle. Cross-validation identified 19 high-risk drugs, including hormonal agents (finasteride, dutasteride, testosterone), antineoplastic drugs (bleomycin, vinblastine, hydroxycarbamide), and antidepressants (citalopram, paroxetine). Finasteride satisfied both dechallenge and rechallenge criteria, providing strong evidence for causality. Signals concentrated in three major categories: genitourinary and sex hormones, dermatological preparations, and antineoplastic agents. Stratified analysis showed that both consumer and healthcare professional reports identified 13 high-risk drugs, with high consistency for major drugs (finasteride, testosterone), confirming result robustness.

**Conclusion:**

This pharmacovigilance study identified 19 high-risk drugs for male infertility across hormonal agents, antineoplastic drugs, and antidepressants. These findings underscore pre-prescription, risk-stratified fertility counseling for reproductive-age males, prioritizing sperm cryopreservation before chemotherapy and considering it selectively for 5α-reductase inhibitors, testosterone, or antidepressants.

## 1 Introduction

Infertility represents a critical global health challenge affecting people of reproductive age, with male factors accounting for a substantial proportion of infertility cases. The Global Burden of Disease study revealed that approximately 55 million men aged 15–49 years were diagnosed with infertility in 2021, reflecting a 74.66% increase since 1990. The age-standardized prevalence rate reached 1354.76 per 100,000 population, marking a 16.90% elevation compared to 1990, which underscores the escalating burden on male reproductive health (Zeng et al., 2025). Given that male infertility is frequently accompanied by profound psychological, familial, and societal consequences, enhanced identification and prevention of causative factors hold paramount significance.

Male reproductive function is influenced by a constellation of genetic and acquired factors. While congenital conditions such as genetic defects and chromosomal abnormalities can precipitate spermatogenic dysfunction, acquired factors including chronic exposure to pesticides and radiation, adverse lifestyle habits such as smoking, alcohol abuse, and high-temperature sauna bathing may also compromise spermatogenesis or sperm maturation processes (Malekasgar and Mombaini, 2008; Garcia-Quevedo et al., 2011; Young et al., 2013; Revonta et al., 2010). Additionally, numerous therapeutic medications have been documented to transiently or persistently suppress spermatogenesis. Direct cellular damage to spermatogonia, Sertoli cells, or Leydig cells, or alterations in the testicular and epididymal microenvironment, may culminate in oligoasthenoteratozoospermia, azoospermia, or even testicular atrophy (Ding et al., 2017). Existing literature indicates that hormonal agents, antineoplastic drugs, certain antidepressants, antiepileptic medications, and immunomodulatory agents may adversely affect male reproductive function. However, these findings predominantly derive from clinical case reports or *in vitro* studies, lacking systematic pharmacovigilance evaluation.

Traditional pharmacological and toxicological assays can indicate the reproductive toxicity of certain medications; nevertheless, clinical trials are constrained by limited sample sizes and abbreviated follow-up durations, making detection of rare or delayed adverse events challenging. Following market authorization, spontaneous reporting systems from real-world settings have emerged as pivotal resources for identifying uncommon adverse events. The United States Food and Drug Administration Adverse Event Reporting System (FAERS) constitutes the world’s largest spontaneous reporting database for adverse drug reactions, encompassing demographic information, pharmacotherapy details, indications, outcomes, and reporting sources. Correspondingly, the European Medicines Agency established the EudraVigilance (EV) database in 2001, aggregating individual case safety reports submitted by regulatory authorities and pharmaceutical companies across European Union member states. Employing dynamic updating and master case consolidation protocols, EV reflects the most current and comprehensive reporting information. The concurrent utilization of these two databases in this study enables cross-validation of signals across diverse populations and reporting systems, thereby enhancing result robustness.

Given the paucity of systematic investigations regarding drug-induced male infertility in existing literature, this study leverages the FAERS database (Q1 2004 to Q2 2025) and EV database (January 2002 to October 2025) to conduct large-scale signal mining of adverse events associated with male infertility. Through this systematic analysis, we aim to identify medications potentially causing male infertility or sperm abnormalities, provide risk alerts for clinical medication practices, and furnish insights for subsequent mechanistic research and drug re-evaluation.

## 2 Methods

### 2.1 Data sources

We employed two major pharmacovigilance databases to conduct disproportionality analysis. Data from the United States Food and Drug Administration Adverse Event Reporting System (FAERS) were obtained through the official website (https://fis.fda.gov/extensions/FPD-QDE-FAERS/FPD-QDE-FAERS.html), encompassing complete ASCII format data packages from the first quarter of 2004 to the second quarter of 2025. Data processing and statistical analyses were performed using SAS version 9.4 software. To enhance result reliability, the European pharmacovigilance database, EV, was concurrently incorporated as a validation data source. Established in 2001, this database was accessed through a formal data access application procedure, retrieving spontaneous reporting data from January 2002 to October 2025. Data sources comprised reports submitted by regulatory authorities and pharmaceutical companies across European Union member states. Notably, the EV database implements a dynamic updating mechanism whereby subsequent reports from the same reporter automatically supersede previous versions, and duplicate cases from multiple reporters are consolidated into a “master case” containing the most comprehensive information, thereby ensuring that each report reflects the most current and complete adverse event data.

### 2.2 Data cleaning and standardization

The FAERS database operates under a spontaneous reporting paradigm, wherein duplicate reports and withdrawn records exist within the dataset. This study rigorously adhered to official FDA guidance for data deduplication: Primary ID, CASE ID, and FDA_DT fields were extracted from the DEMO table and subjected to three-tier sorting by CASE ID, FDA_DT, and Primary ID. For identical CASE IDs, records with the maximum FDA_DT value (most recent date) were retained; when both CASE ID and FDA_DT were identical, the record with the maximum Primary ID value was preserved. Commencing from the first quarter of 2019, withdrawn reports were further excluded based on deletion lists accompanying each quarterly data package. Adverse event nomenclature was standardized using the Medical Dictionary for Regulatory Activities version 28.0 (MedDRA 28.0), with coding conducted at the Preferred Term and System Organ Class hierarchical levels. Drug nomenclature underwent standardization using the World Health Organization Drug Dictionary, March 2025 edition (WHO-DD, March 2025). Given that routine MedDRA updates in March and September annually may precipitate terminological hierarchical adjustments, this study employed the most recent version to uniformly revalidate and remap all historical data, ensuring analytical consistency and comparability.

### 2.3 Study population and target event definition

We first constructed deduplicated patient-level datasets from both FAERS (following the FDA algorithm) and EV (following EMA’s master case consolidation protocol). Male-infertility–associated events were then identified using a predefined set of 13 MedDRA Preferred Terms (Table 1), which comprehensively covered quantitative and qualitative sperm abnormalities, spermatogenic disorders, clinical infertility diagnosis, and reproductive organ pathology. For both databases, adverse event reports were extracted when the reported Preferred Term exactly matched any of the terms in Table 1. We employed an exact-match strategy to ensure precision and reproducibility. Analysis was restricted to reports where the study drug was coded as “Primary Suspect (PS)” in FAERS or “Suspected” in EV, and where sex was identified as male. This systematic approach identified 1,955 unique cases from FAERS (corresponding to 2,173 drug-event records, as some cases reported multiple suspected drugs) and 1,384 cases from EV. All included cases were male patients.

**TABLE 1:** Screening list of MedDRA terms related to male infertility-associated adverse events.

MedDRA preferred term code	MedDRA preferred term
10050208	Teratospermia
10030300	Oligospermia
10067162	Asthenospermia
10041493	Spermatogenesis abnormal
10021929	Infertility male
10079294	Oligoasthenoteratozoospermia
10080320	Oligoasthenozoospermia
10074268	Reproductive toxicity
10074729	Necrospermia
10003495	Aspermia
10003883	Azoospermia
10049572	Testicular necrosis
10043298	Testicular atrophy

### 2.4 Signal detection criteria

The disproportionality analysis was restricted to male reports only. For each drug-male infertility pair, the reporting frequency was compared against all other drug-adverse event combinations reported for male patients in the respective database. A four-fold contingency table was constructed (Supplementary Table S1). This study employed disproportionality analysis methods including the reporting odds ratio (ROR), proportional reporting ratio (PRR), Bayesian confidence propagation neural network (BCPNN), and multi-item gamma Poisson shrinker (MGPS) to investigate the associations between selected drugs and male infertility events. The calculation formulas and thresholds are shown in Supplementary Table S2. The basic principle of disproportionality analysis is to estimate the ratio of the actual number of adverse drug reactions associated with a specific drug reported to the expected number or to the number of other adverse reactions caused by other drugs. If the ratio reaches a certain degree (disproportionality), an association exists between the drug and the adverse reaction. This study adopted a combined approach using ROR, PRR, BCPNN, and MGPS methods to reduce bias from false-positive signals. A positive signal was generated only when all four analytical methods met their respective thresholds simultaneously: number of adverse event reports ≥3, lower limit of 95% CI of ROR >1; PRR ≥2 and χ^2^ ≥ 4; IC025 > 0; EBGM05 > 2. The ROR value was used to assess the strength of signal association with the drug, with higher ROR values indicating stronger associations between the selected drug and male infertility.

### 2.5 Stratified analysis and sensitivity analysis

To comprehensively evaluate signal robustness, we conducted stratified analyses using all FAERS (n = 1,955) and EV (n = 1,384) cases (Section 2.3), and a FAERS dechallenge/rechallenge sensitivity analysis (not as inclusion criteria). In the FAERS DRUG table, DECHAL denotes event improvement after discontinuation and RECHAL denotes recurrence after re-administration; codes are Y (positive), N (negative), U (unknown), and D (not applicable). Using primary suspect drugs (ROLE_COD = PS) and the main-analysis cleaning/deduplication rules, we built DECHAL = Y and RECHAL = Y subsets and computed 2 × 2 disproportionality metrics (ROR, PRR, χ^2^, BCPNN-IC, MGPS-EBGM) applying the primary thresholds (a≥3; ROR025 > 1; PRR≥2 and χ^2^ ≥ 4; IC025 > 0; EBGM05 > 2).

Stratification by reporter identity was performed as follows: in the FAERS database, Consumer and Lawyer categories were classified as consumers, while Physician, Pharmacist, and Other Health-Professional categories were classified as healthcare professionals; in the EV database, Non Healthcare Professional was classified as consumers and Healthcare Professional as healthcare professionals, with the aforementioned six criteria applied for signal detection in each stratum. The Anatomical Therapeutic Chemical (ATC) Classification System at the first level was employed to evaluate association strength across different therapeutic drug categories. Given that certain drugs in the EV database could not be accurately mapped to ATC codes, this analysis was conducted exclusively using the FAERS database. For reports in the FAERS database with available time-to-event information, Kaplan-Meier methodology was employed to construct cumulative distribution of time-to-onsets, with temporal distribution differences compared across report seriousness strata (Wilcoxon test). To enhance signal reliability, a cross-validation strategy was adopted, whereby the intersection of detection results from both FAERS and EV databases was designated as high-confidence signals, thereby mitigating inherent biases of individual databases. All statistical analyses were performed using SAS version 9.4. Signal strength was visualized using forest plots with logarithmic scale on the horizontal axis and error bars representing 95% confidence intervals.

### 2.6 Ethical statement

This study utilized publicly accessible data from the FAERS and EV databases, both of which contain fully anonymized and de-identified patient information. Formal ethical approval and informed consent were not required for this study. This exemption was granted based on the following considerations: (1) all data originated from publicly accessible databases and contained no personally identifiable information; (2) these databases were established under appropriate regulatory oversight to ensure patient privacy protection; (3) our analysis posed no risk to individual research subjects, as we had no access to identifying information.

## 3 Results

### 3.1 Demographic characteristics and baseline information of reported cases

**FIGURE 1:**
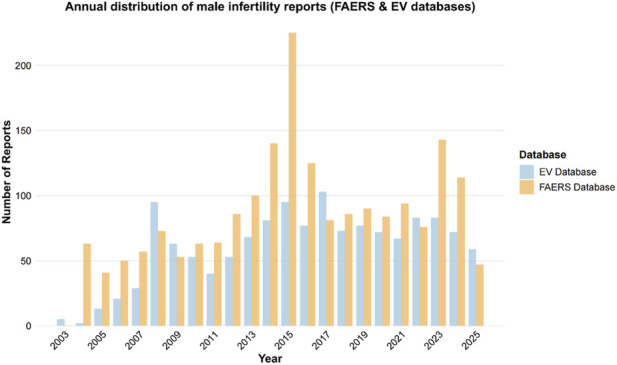
Annual distribution of male infertility-related reports in the FAERS and EV databases.

We incorporated 1,955 and 1,384 male infertility-associated adverse event reports from the FAERS and EV databases, respectively, with all cases pertaining to male patients. The reports from both databases exhibited distinct annual distribution trends (Figure 1). In the FAERS database, cases with available age information demonstrated an age range spanning 0–95 years, with a median age of 35 years (interquartile range [IQR] 29–49 years) and a mean age of 39.78 years. The age composition was predominantly composed of reproductive-age young adults aged 18–44 years (722 cases), followed by middle-aged individuals aged 45–64 years (209 cases) and elderly patients aged ≥65 years (118 cases). Regarding reporting sources, consumer reports were most prevalent (833 cases), followed by physician reports (575 cases), with the remainder consisting of other health professionals (253 cases), pharmacists (142 cases), lawyers (35 cases), and unspecified sources (117 cases) (Table 2). In the EV database, all 1,384 male infertility-associated adverse event reports originated from male patients. The age distribution was predominantly composed of adults aged 18–64 years (756 cases). Concerning reporter types, healthcare professional reports constituted the predominant proportion (917 cases), followed by non-healthcare professional reports (449 cases). Geographically, reports primarily originated from European Economic Area countries (760 cases) (Supplementary Table S3).

**TABLE 2:** Demographic characteristics and clinical baseline data of cases reported in the FAERS database.

Characteristics	n (%)
Sex	
Male	1955 (100.0)
Age	
<18	52 (2.66)
18-44	722 (36.93)
45-64	209 (10.69)
≥65	118 (6.04)
Not specified	854 (43.68)
Reporter	
Consumer	833 (42.61)
Lawyer	35 (1.79)
Not specified	117 (5.98)
Other health-professional	253 (12.94)
Pharmacist	142 (7.26)
Physician	575 (29.41)

### 3.2 Time-to-event analysis from drug administration to male infertility onset

**FIGURE 2:**
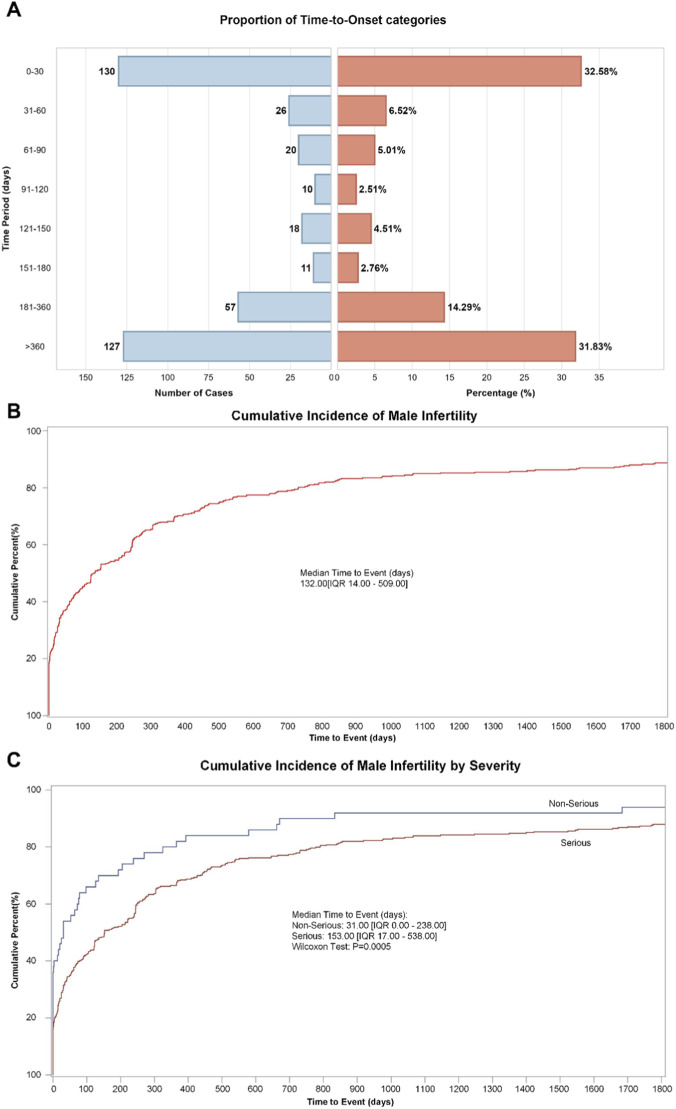
Temporal distribution characteristics from drug administration to male infertility adverse event onset. **(A)** Temporal segmental distribution and proportional composition. **(B)** Cumulative distribution of time-to-onset (Kaplan-Meier analysis). **(C)** Cumulative distribution of time-to-onsets stratified by seriousness.

Among the total 1,955 reports, 399 cases documented time-to-event information from first medication administration to first occurrence of the male infertility adverse event. While this proportion reflects the incomplete temporal documentation characteristic of spontaneous reporting systems, the available data revealed interpretable temporal patterns. The temporal segmental distribution (Figure 2A) exhibited a distinct bimodal pattern: early reports (0–30 days, 130 cases) and late reports (>360 days, 127 cases) collectively accounted for 64.41%, whereas intermediate-period reports (31–360 days) were relatively fewer (142 cases). The cumulative distribution of time-to-onset (Figure 2B) demonstrated a pattern characterized by rapid early accumulation followed by gradual plateau, with a median time of 132 days (IQR 14–509 days). Specifically, among the 399 cases with documented time-to-event data, 45% of events occurred within 100 days from first medication administration, 56% within 200 days, followed by decelerated accumulation, with the curve gradually reaching a plateau phase after 700–900 days. Stratified analysis by seriousness revealed significant temporal distribution differences (Wilcoxon test, *P* = 0.0005). Non-serious reports exhibited a shorter median time (31 days, IQR 0–238 days), with approximately two-thirds occurring within 100 days from first medication administration, whereas serious reports demonstrated a markedly prolonged median time (153 days, IQR 17–538 days), with more dispersed temporal distribution and extended cumulative duration (Figure 2C).

### 3.3 High-risk drug identification through cross-database validation

This study conducted signal detection in both FAERS and EV databases independently, employing multiple analytical methods in combination. Results demonstrated that the FAERS database identified 35 drugs meeting the criteria (cumulative reports: 1,205 cases), while the EV database identified 61 drugs meeting the criteria (cumulative reports: 1,116 cases). To enhance signal reliability, this study implemented a cross-database validation strategy, ultimately confirming 19 drugs with positive signals detected in both databases (Table 3).

**TABLE 3:** Results of cross-database validation of drug signal detection.

Drug name	FAERS						EV					
	n	ROR (95% CI)	PRR (95% CI)	χ^2^	IC(IC025)	EB (EB05)	n	ROR (95% CI)	PRR (95% CI)	χ^2^	IC(IC025)	EB (EB05)
Bleomycin	14	57.29 (33.82,97.06)	56.93 (33.72,96.13)	764.44	5.82 (2.84)	56.57 (33.39)	24	24.91 (16.65,37.27)	24.83 (16.62,37.11)	542.85	4.62 (3.08)	24.56 (16.42)
Carbamazepine	26	5.83 (3.96,8.58)	5.83 (3.96,8.58)	102.72	2.53 (1.73)	5.77 (3.92)	19	4.05 (2.58,6.36)	4.04 (2.57,6.35)	43.16	2.01 (1.15)	4.02 (2.56)
Chorionic gonadotrophin	7	46.82 (22.25,98.52)	46.58 (22.22,97.64)	311.25	5.54 (1.77)	46.43 (22.07)	3	52.11 (16.73,162.31)	51.78 (16.75,160.08)	149.20	5.69 (0.47)	51.71 (16.60)
Citalopram	26	7.13 (4.84,10.50)	7.13 (4.84,10.49)	135.36	2.82 (1.97)	7.05 (4.79)	13	4.07 (2.36,7.03)	4.07 (2.36,7.02)	29.94	2.02 (0.96)	4.05 (2.35)
Doxorubicin	25	7.12 (4.80,10.56)	7.11 (4.80,10.55)	129.81	2.82 (1.94)	7.04 (4.75)	25	4.21 (2.84,6.25)	4.21 (2.84,6.24)	60.45	2.06 (1.32)	4.17 (2.81)
Dutasteride	22	12.74 (8.37,19.39)	12.72 (8.36,19.36)	235.22	3.66 (2.46)	12.60 (8.28)	16	14.64 (8.95,23.96)	14.62 (8.94,23.90)	201.51	3.86 (2.31)	14.52 (8.87)
Etoposide	15	7.11 (4.28,11.81)	7.10 (4.28,11.80)	78.13	2.82 (1.63)	7.06 (4.25)	28	5.04 (3.47,7.31)	5.03 (3.47,7.31)	89.34	2.32 (1.59)	4.98 (3.43)
Finasteride	469	73.90 (66.70,81.87)	73.43 (66.31,81.31)	26,276.9	5.85 (5.54)	57.79 (52.17)	288	60.07 (53.03,68.04)	59.68 (52.73,67.55)	14,365.3	5.69 (5.28)	51.72 (45.66)
Hydroxycarbamide	13	24.04 (13.92,41.49)	23.97 (13.91,41.33)	284.53	4.58 (2.40)	23.84 (13.81)	34	31.39 (22.35,44.08)	31.27 (22.30,43.85)	980.44	4.94 (3.56)	30.79 (21.92)
Leuprorelin	88	5.75 (4.65,7.12)	5.75 (4.64,7.11)	331.23	2.47 (2.09)	5.56 (4.49)	27	6.34 (4.34,9.28)	6.34 (4.34,9.27)	119.92	2.65 (1.85)	6.27 (4.29)
Medroxyprogesterone	4	58.09 (21.71,155.42)	57.72 (21.71,153.45)	222.58	5.85 (0.93)	57.62 (21.54)	3	41.69 (13.39,129.75)	41.48 (13.41,128.32)	118.35	5.37 (0.45)	41.42 (13.31)
Minoxidil	17	3.30 (2.04,5.31)	3.29 (2.04,5.31)	26.95	1.71 (0.86)	3.28 (2.03)	11	9.50 (5.25,17.20)	9.49 (5.25,17.16)	83.18	3.24 (1.64)	9.45 (5.22)
Paroxetine	27	3.94 (2.69,5.76)	3.94 (2.69,5.75)	58.44	1.96 (1.27)	3.90 (2.67)	18	3.28 (2.06,5.22)	3.28 (2.06,5.22)	28.32	1.71 (0.88)	3.26 (2.05)
Sulfasalazine	11	15.52 (8.58,28.08)	15.49 (8.57,28.01)	148.41	3.95 (1.97)	15.42 (8.52)	11	4.32 (2.39,7.81)	4.31 (2.39,7.80)	27.86	2.10 (0.92)	4.30 (2.38)
Tamsulosin	14	3.57 (2.11,6.03)	3.57 (2.11,6.03)	25.68	1.83 (0.85)	3.55 (2.10)	22	5.16 (3.39,7.85)	5.15 (3.39,7.84)	72.92	2.35 (1.51)	5.11 (3.36)
Testosterone	211	23.09 (20.03,26.61)	23.03 (19.99,26.54)	4015.88	4.39 (4.05)	20.89 (18.13)	71	12.17 (9.60,15.42)	12.15 (9.59,15.39)	702.50	3.56 (3.01)	11.78 (9.30)
Triptorelin	5	8.58 (3.56,20.63)	8.57 (3.56,20.60)	33.35	3.10 (0.74)	8.55 (3.55)	7	12.16 (5.79,25.55)	12.14 (5.78,25.48)	71.34	3.60 (1.32)	12.10 (5.76)
Valproic acid	49	6.40 (4.82,8.49)	6.40 (4.82,8.49)	218.02	2.65 (2.09)	6.27 (4.73)	70	6.06 (4.77,7.69)	6.05 (4.77,7.68)	285.62	2.56 (2.11)	5.89 (4.64)
Vinblastine	15	371.39 (221.20,623.54)	356.65 (216.82,586.66)	5283.66	8.47 (3.20)	354.19 (210.96)	21	31.83 (20.69,48.97)	31.71 (20.65,48.69)	618.43	4.97 (3.10)	31.40 (20.41)

**FIGURE 3:**
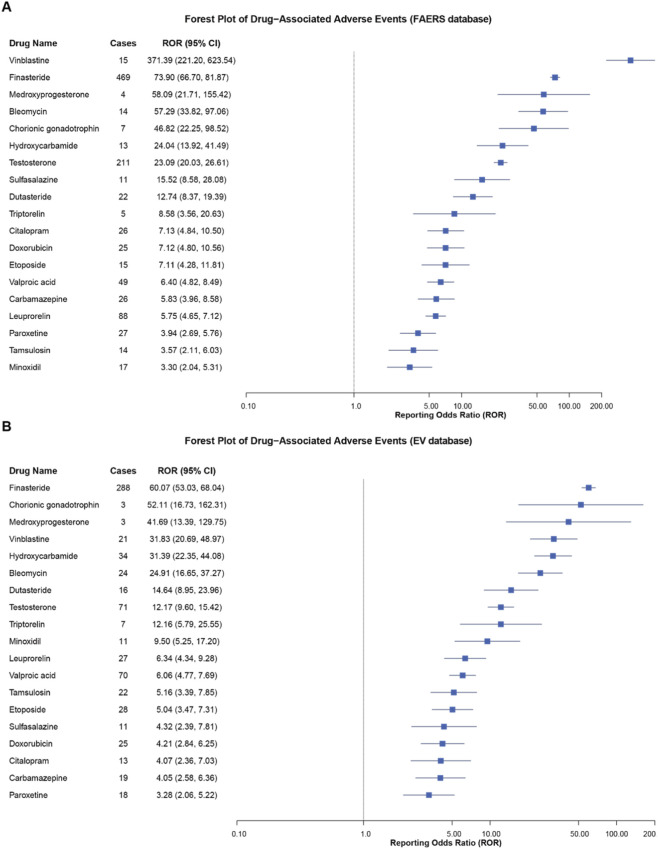
Forest plots of signal strength for 19 high-risk drugs validated through cross-database verification. **(A)** Signal detection results from the FAERS database. **(B)** Signal detection results from the EV database. Drugs are ranked in descending order by ROR values, with the horizontal axis displayed on a logarithmic scale and error bars representing 95% confidence intervals.

Forest plots visualized the signal strength of each drug and their 95% confidence intervals (CIs), with drugs ranked in descending order by ROR values. In the FAERS database, vinblastine demonstrated the highest ROR value (371.39, 95% CI 221.20-623.54), followed by finasteride (ROR = 73.90) and hydroxycarbamide (ROR = 24.04) (Figure 3A). In the EV database, finasteride exhibited the highest ROR value (60.07, 95% CI 53.03-68.04), followed by chorionic gonadotrophin (ROR = 52.11) and medroxyprogesterone acetate (ROR = 41.69) (Figure 3B).

### 3.4 Association analysis between different therapeutic drug categories and male infertility

To systematically evaluate the associations between different therapeutic drug categories and male infertility-related adverse events, this study employed the ATC Classification System to categorize drugs in the FAERS database, calculating case counts and signal strength for each therapeutic category. Given that certain drugs in the EV database could not be accurately mapped to ATC classifications, this analysis was conducted exclusively using the FAERS database. We mapped all drug-event records to their corresponding ATC first-level classifications, encompassing 14 therapeutic categories (Table 4). Since certain drugs are classified under multiple ATC categories, the 2,173 drug-event records generated 3,248 drug-event-ATC classification pairs for analysis. Regarding the distribution across categories, drugs acting on the genitourinary system and sex hormones exhibited the highest number of records (n = 851), followed by dermatological preparations (n = 627) and antineoplastic and immunomodulating agents (n = 573). Additionally, considerable numbers of records were observed for nervous system drugs (n = 443), cardiovascular system drugs (n = 188), and systemic hormonal preparations excluding sex hormones and insulins (n = 143).

**TABLE 4:** Results of drug signal detection for male infertility based on ATC classification.

ATC classification	n	ROR (95% CI)	PRR (95% CI)	χ^2^	IC(IC025)	EB (EB05)
Alimentary tract and metabolism	84	0.24 (0.20,0.30)	0.24 (0.20,0.30)	189.02	−1.87 (-2.18)	0.27 (0.22)
Blood and blood forming organs	25	0.16 (0.11,0.24)	0.16 (0.11,0.24)	107.84	−2.55 (-3.07)	0.17 (0.12)
Cardiovascular system	188	0.76 (0.65,0.88)	0.76 (0.65,0.88)	13.11	−0.36 (-0.58)	0.78 (0.67)
Dermatologicals	627	3.56 (3.24,3.90)	3.56 (3.24,3.90)	819.38	1.49 (1.36)	2.82 (2.57)
Genitourinary system and sex hormones	851	9.76 (8.95,10.64)	9.75 (8.95,10.63)	4067.64	2.66 (2.53)	6.33 (5.80)
Systemic hormonal preparations, excl. sex hormones and insulins	143	1.83 (1.54,2.17)	1.83 (1.54,2.17)	50.19	0.83 (0.57)	1.77 (1.50)
Antiinfectives for systemic use	72	0.43 (0.34,0.54)	0.43 (0.34,0.54)	53.42	−1.16 (-1.50)	0.45 (0.35)
Antineoplastic and immunomodulating agents	573	0.67 (0.61,0.74)	0.67 (0.61,0.74)	68.63	−0.40 (-0.54)	0.76 (0.69)
Musculo-skeletal system	82	0.89 (0.72,1.11)	0.89 (0.72,1.11)	1.04	−0.16 (-0.48)	0.90 (0.72)
Nervous system	443	1.00 (0.90,1.11)	1.00 (0.90,1.11)	0.01	0.01 (-0.15)	1.00 (0.90)
Antiparasitic products, insecticides and repellents	5	0.78 (0.32,1.87)	0.78 (0.32,1.87)	0.32	−0.36 (-1.49)	0.78 (0.32)
Respiratory system	47	0.28 (0.21,0.37)	0.28 (0.21,0.37)	85.84	−1.76 (-2.16)	0.29 (0.22)
Sensory organs	100	0.62 (0.51,0.76)	0.62 (0.51,0.76)	21.63	−0.64 (-0.93)	0.64 (0.52)
Various	8	0.16 (0.08,0.33)	0.16 (0.08,0.33)	33.88	−2.58 (-3.40)	0.17 (0.08)

### 3.5 Positive signal analysis of dechallenge and rechallenge

Dechallenge/rechallenge analysis was employed to evaluate resolution/disappearance of male infertility following drug discontinuation and recurrence upon re-administration. This study identified 12 drugs meeting positive dechallenge signal criteria (Table 5), among which finasteride exhibited the highest case count (n = 45), while dutasteride and valganciclovir, despite lower case numbers, demonstrated elevated signal strength indicators. Applying identical signal detection criteria, 2 drugs were identified as meeting positive rechallenge signal criteria (Table 5), specifically finasteride and etanercept. Notably, finasteride simultaneously satisfied both dechallenge and rechallenge criteria, providing evidentiary support for its association with male infertility.

**TABLE 5:** List of drugs with positive dechallenge and rechallenge signals associated with male infertility.

Drug name	n	ROR (95% CI)	PRR (95% CI)	χ^2^	IC(IC025)	EB (EB05)
Positive signal dechallenge drug						
Finasteride	45	106.29 (75.90,148.84)	105.61 (75.55,147.65)	3529.08	6.32 (4.40)	80.17 (57.25)
Testosterone	15	39.13 (23.06,66.39)	39.02 (23.03,66.11)	510.68	5.17 (2.74)	35.94 (21.18)
Escitalopram	9	17.52 (8.96,34.24)	17.50 (8.96,34.17)	133.18	4.06 (1.76)	16.69 (8.54)
Venlafaxine	4	6.81 (2.53,18.36)	6.81 (2.53,18.34)	19.40	2.74 (0.33)	6.69 (2.48)
Atomoxetine	4	8.88 (3.30,23.93)	8.88 (3.30,23.90)	27.36	3.12 (0.47)	8.71 (3.23)
Leuprorelin	3	13.51 (4.31,42.29)	13.49 (4.31,42.20)	34.14	3.73 (0.25)	13.29 (4.24)
Minocycline	3	23.62 (7.54,73.99)	23.58 (7.54,73.70)	63.81	4.54 (0.36)	23.21 (7.41)
Dutasteride	3	41.21 (13.14,129.20)	41.08 (13.15,128.32)	115.42	5.34 (0.43)	40.43 (12.89)
Valganciclovir	3	49.29 (15.72,154.60)	49.11 (15.73,153.35)	139.10	5.59 (0.45)	48.33 (15.41)
Citalopram	3	7.00 (2.23,21.90)	6.99 (2.23,21.88)	15.16	2.79 (0.02)	6.90 (2.20)
Verapamil	3	30.58 (9.76,95.83)	30.51 (9.76,95.35)	84.24	4.91 (0.40)	30.03 (9.58)
Spironolactone	3	10.72 (3.42,33.56)	10.71 (3.42,33.50)	25.98	3.40 (0.18)	10.55 (3.37)
Positive signal rechallenge drug						
Finasteride	5	40.95 (15.34,109.35)	40.75 (15.32,108.34)	155.11	5.04 (1.06)	32.80 (12.28)
Etanercept	3	18.67 (5.58,62.48)	18.63 (5.58,62.15)	44.05	4.05 (0.20)	16.51 (4.94)

### 3.6 Comparison of drug signals between consumer and healthcare professional reports

**FIGURE 4:**
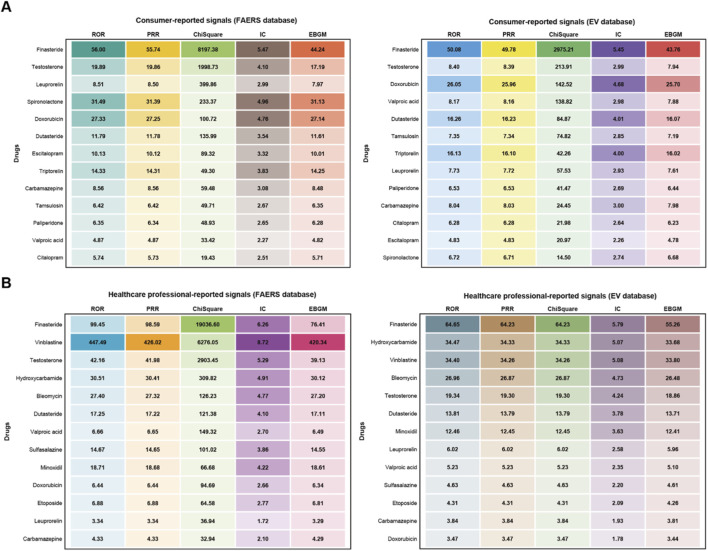
Drug signal detection results based on reporter type. **(A)** Heatmap of drug signal strength from consumer reports. **(B)** Heatmap of drug signal strength from healthcare professional reports.

To investigate the impact of different reporting sources on drug signal detection outcomes, this study conducted stratified analyses of reporters in both FAERS and EV databases. Based on reporter identity, reports were categorized into consumer reports and healthcare professional reports, with identical signal detection criteria applied for stratified screening. Among consumer reports, both FAERS and EV databases identified 13 drugs meeting signal detection criteria (Figure 4A). The drugs with the highest case counts in both databases were finasteride (FAERS: n = 193, EV: n = 71) and testosterone (FAERS: n = 130, EV: n = 35). Additionally, dutasteride reports were more prevalent in the FAERS database (n = 21), whereas valproic acid reports were relatively more numerous in the EV database (n = 21). Among healthcare professional reports, both FAERS and EV databases similarly identified 13 drugs meeting signal detection criteria (Figure 4B). Finasteride ranked first in case counts in both databases (FAERS: n = 255, EV: n = 216), followed by testosterone (FAERS: n = 78, EV: n = 36). Leuprorelin reports were more abundant in the FAERS database (n = 66), while hydroxycarbamide reports were relatively more numerous in the EV database (n = 34).

## 4 Discussion

This study conducted systematic signal mining for drug-associated male infertility based on the United States FAERS database and European EV database, providing crucial safety evidence for clinical medication practices through rigorous data cleaning, multi-indicator combined screening, and dechallenge/rechallenge analyses to validate drug-adverse event relationships. The study identified 1,955 male infertility-related adverse event reports in the FAERS database, with a median patient age of 35 years (IQR 29.0–49.0), among which reproductive-age males aged 18–44 years constituted 36.93%, an age distribution highly concordant with the peak incidence age range for male infertility globally. Notably, among the 399 reports with available medication timing information, the median time to event onset was 132 days from first medication administration (IQR 14–509), with approximately 45% of events occurring within 100 days from first medication administration. This temporal window demonstrates remarkable biological congruence with the spermatogenic cycle (approximately 74 days) and epididymal maturation process (approximately 12–14 days), suggesting that drug effects on spermatogenesis may manifest following one complete spermatogenic cycle. Beyond spermatogenic timing, sperm motility can also be regulated by components of the seminal milieu, including seminal plasma extracellular vesicles that deliver bioactive signals to spermatozoa (Liu et al., 2026).

It is important to acknowledge that time-to-event data were available for only 20.4% of cases, meaning that our temporal observations are based on a subset with documented timing information. While this limited completeness represents an inherent constraint of spontaneous reporting databases, the observed temporal pattern demonstrates strong biological plausibility, with the median onset at 132 days aligning remarkably with the known duration of spermatogenesis and epididymal maturation. These temporal insights, though derived from incomplete data, provide hypothesis-generating observations and biologically grounded guidance for clinical monitoring timeframes. Prospective studies with systematic temporal documentation are warranted to validate and refine these preliminary temporal associations.

The time-to-event analysis revealed a bimodal distribution with peaks at 0–30 days and >360 days, suggesting mechanistically distinct pathways across drug classes. The early peak likely reflects drugs causing rapid endocrine disruption or functional sperm impairment. Exogenous testosterone immediately suppresses the hypothalamic-pituitary-gonadal axis, with hormonal contraception studies reporting mean time to azoospermia of approximately 120 days (Crosnoe et al., 2013). Selective serotonin reuptake inhibitors (SSRIs) demonstrate similarly rapid effects, with paroxetine increasing sperm DNA fragmentation within 4 weeks and morphological changes emerging after approximately 3 months consistent with spermatogenic cycle duration (Tanrikut et al., 2010; Xu et al., 2022). The late peak likely reflects cumulative gonadotoxicity (chemotherapeutic agents causing prolonged germ cell damage with recovery extending months to years) and delayed clinical recognition (endocrine disruptors producing insidious declines detected only during fertility evaluation). However, male infertility is often recognized only after prolonged unsuccessful conception attempts, meaning extended time-to-event values may reflect diagnostic and reporting latency rather than purely biological onset. These temporal patterns, though derived from incomplete data (20.4% documentation rate), warrant targeted investigation through prospective studies with systematic temporal monitoring.

**FIGURE 5:**
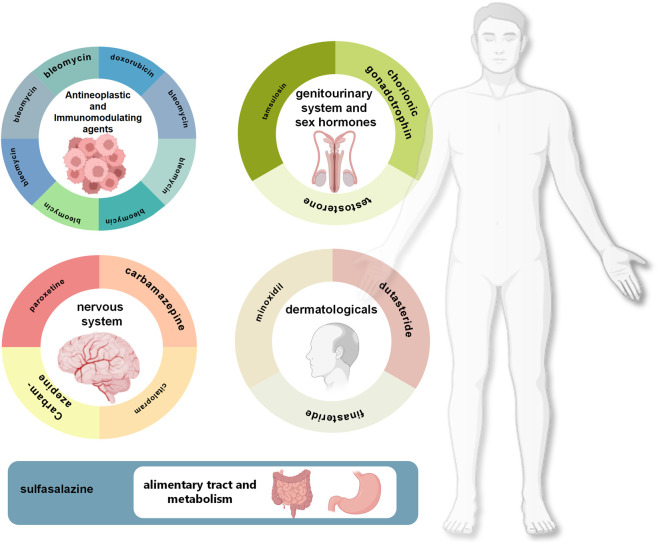
ATC classification distribution and representative drugs associated with high-risk male infertility.

Through cross-database validation, this study ultimately confirmed 19 high-confidence risk drugs, encompassing multiple therapeutic domains including hormonal agents, antineoplastic drugs, and antidepressants. Analysis by ATC first-level classification revealed that signals were predominantly concentrated in the following categories: genitourinary system and sex hormones, dermatological preparations, antineoplastic and immunomodulating agents, nervous system drugs, and alimentary tract and metabolism drugs (Figure 5). Stratified analysis by reporter type unveiled differential concerns regarding drug reproductive toxicity across populations: consumer reports predominantly focused on alopecia treatment drugs (finasteride, dutasteride), testosterone supplements, and antidepressants, reflecting patient awareness of reproductive adverse effects associated with routine medications, whereas healthcare professional reports concentrated more extensively on chemotherapeutic agents and immunosuppressants, embodying heightened attention to fertility preservation in oncological and immunological disease patients.

This reporting source heterogeneity suggests that differentiated risk communication and shared decision-making strategies should be tailored to distinct drug categories and clinical contexts. For elective medications for non-life-threatening conditions (e.g., finasteride/dutasteride for alopecia, testosterone for hypogonadism, antidepressants for psychiatric disorders), pre-prescription counseling should explicitly address fertility implications, reversibility timelines, and therapeutic alternatives, enabling patients to weigh reproductive risks against treatment benefits. For essential life-saving therapies (e.g., chemotherapeutic agents for malignancies, immunosuppressants for severe autoimmune diseases), where treatment deferral is not feasible, risk communication should prioritize proactive fertility preservation strategies (sperm cryopreservation before treatment initiation) alongside comprehensive risk-benefit disclosure. For testosterone replacement therapy, counseling should differentiate between physiological replacement in hypogonadal patients (where fertility preservation may be achievable through concurrent gonadotropin therapy) versus supraphysiological use in non-medical contexts (where reproductive suppression is predictable). This risk-stratified, category-specific communication framework ensures that reproductive-age males receive contextually appropriate information—emphasizing treatment alternatives for elective medications, fertility preservation for essential therapies, and nuanced counseling for hormonal interventions—thereby supporting informed decisions aligned with individual therapeutic needs and reproductive goals.

Finasteride and dutasteride, first-line therapies for benign prostatic hyperplasia and androgenetic alopecia, demonstrated robust signals. These 5α-reductase inhibitors reduce serum DHT by 70%–90% (Marihart et al., 2005; Roehrborn et al., 2004). Finasteride uniquely satisfied both dechallenge and rechallenge criteria, with documented symptom resolution post-discontinuation and recurrence upon re-administration, providing compelling causality evidence. Case reports confirm significant reductions in sperm DNA fragmentation and improvements in concentration/motility following discontinuation (Gude, 2011). However, prolonged use (>18 months) can significantly diminish semen parameters, with patients treated ≥20 months potentially experiencing persistent impairment despite discontinuation (Kim et al., 2025). Minoxidil, commonly co-prescribed with finasteride for alopecia, also generated signals in our analysis. Systematic reviews suggest that both finasteride and minoxidil may induce hormonal and oxidative stress-related testicular alterations, primarily demonstrated in animal models (Santana et al., 2023), although human evidence linking topical minoxidil to male infertility remains limited and primarily associated with high oral doses (Hui et al., 2022). For males with fertility plans, clinicians should prioritize low-dose, short-duration regimens and consider pre-treatment sperm cryopreservation.

Exogenous testosterone showed significant signals in both databases. Through negative feedback suppression of the HPG axis, testosterone reduces GnRH/LH/FSH secretion, causing ∼94% decline in intratesticular testosterone and near-complete spermatogenic arrest (Patel et al., 2019; Song et al., 2019; Lee and Ramasamy, 2018). Clinical evidence confirms this: a case series of eight men on testosterone therapy developed azoospermia/severe oligozoospermia, with sperm concentration and FSH recovering to baseline after a mean 8.5 months post-discontinuation (Bang et al., 2013), indicating reversibility albeit with extended recovery. Prior to prescribing testosterone to reproductive-age males, clinicians must evaluate fertility plans, endogenous testosterone levels, and testicular reserve, while offering pre-treatment sperm cryopreservation to prevent irreversible loss.

GnRH agonists (leuprorelin, triptorelin) demonstrated male infertility signals consistent with their established mechanism of action. These agents induce pituitary GnRH receptor desensitization through continuous stimulation, resulting in profound suppression of LH/FSH secretion and subsequent hypogonadotropic hypogonadism—a phenomenon clinically exploited for androgen deprivation therapy in prostate cancer (Merseburger and Hupe, 2016; Crawford and Phillips, 2011). Long-term GnRH agonist administration causes significant declines in mean sperm counts, with some patients developing azoospermia or severe oligozoospermia (Bhasin et al., 1987). This iatrogenic reproductive suppression is well-recognized in reproductive medicine as a predictable consequence of medically induced hypogonadism (Velez and Ohlander, 2021). Medroxyprogesterone acetate, particularly in depot formulations, similarly produces fertility suppression as an expected pharmacologic effect; signals detected for this agent likely reflect this anticipated mechanism rather than unanticipated toxicity. Human chorionic gonadotropin’s inclusion in our signal set requires careful interpretation, as its therapeutic use for treating hypogonadism-related infertility likely reflects indication bias rather than actual drug toxicity. Controlled prospective studies are essential to disentangle treatment effects from indication confounding for these agents.

Regarding psychiatric medications, this study detected signals for multiple antidepressant drugs, including SSRIs such as escitalopram and citalopram, as well as the serotonin-norepinephrine reuptake inhibitor (SNRI) venlafaxine. Meta-analyses show SSRIs significantly reduce sperm morphology, concentration, and motility while increasing DNA fragmentation, with effects typically emerging within 3 months—consistent with our observed 100-day peak (Xu et al., 2022). However, venlafaxine signals warrant caution given that depression itself may compromise sperm quality through oxidative stress and hormonal imbalance, and polypharmacy is common. Animal studies show dose-dependent effects (Saleem et al., 2020). Reproductive risk assessment must differentiate direct drug effects from disease impacts, with individualized treatment selection and periodic semen monitoring for patients with fertility intentions.

Among nervous system medications, antiepileptic drugs also generated signals warranting clinical attention. Carbamazepine monotherapy in male epilepsy patients has been associated with altered semen quality, including abnormalities in concentration, morphology, and motility (Asadi-Pooya et al., 2015). Valproic acid demonstrates more robust evidence for reproductive toxicity: clinical studies report reductions in sperm count, motility, and increased teratozoospermia, with partial reversibility observed following discontinuation in some patients (Tallon et al., 2021). Systematic reviews synthesizing clinical and animal data consistently suggest associations between VPA and impaired male reproductive parameters (Asghar et al., 2024). Given that epilepsy management often requires long-term therapy, reproductive-age males initiating antiepileptic drugs should receive counseling regarding potential fertility impacts, with consideration of alternative agents when clinically feasible and periodic semen analysis monitoring during treatment.

Among the 19 cross-validated drugs, five (26%) were antineoplastic agents (bleomycin, doxorubicin, etoposide, hydroxycarbamide, vinblastine), consistent with established reproductive medicine knowledge regarding spermatogenic cell sensitivity to chemotherapy. Alkylating agents (cyclophosphamide, busulfan, chlorambucil, mechlorethamine) and platinum compounds induce prolonged/permanent azoospermia at high doses but typically permit recovery within 1–3 years at lower doses (Meistrich, 2009). Vinblastine (microtubule inhibitor) disrupts mitosis, causing spermatogenic cell arrest and transient azoospermia/oligozoospermia. Hydroxycarbamide (ribonucleotide reductase inhibitor) shows specific toxicity to proliferating spermatogonia (Berthaut et al., 2008; DeBaun, 2014). Doxorubicin, an anthracycline antibiotic, induces reproductive toxicity through multiple mechanisms including testicular mass reduction, histologic damage, oligozoospermia, and sperm morphological abnormalities (Mohan et al., 2021); however, anthracyclines generally do not cause persistent azoospermia when administered alone but may produce additive gonadotoxic effects in combination regimens (Meistrich, 2009). Bleomycin, though human data are limited, causes sperm DNA fragmentation and testicular damage in animal models (De Almeida et al., 2024). For young cancer patients, prioritize low reproductive toxicity protocols with pre-treatment sperm cryopreservation and post-treatment monitoring per international guidelines.

Sulfasalazine, used in inflammatory bowel disease management, demonstrated male infertility signals concordant with prior literature. Clinical studies in IBD patients report oligozoospermia, reduced motility, and increased teratozoospermia associated with sulfasalazine therapy (O’Moráin et al., 1984), attributed to its effects on folate metabolism and oxidative stress pathways. Notably, these effects are typically reversible upon discontinuation or switching to alternative 5-aminosalicylic acid derivatives. Tamsulosin, an α1-adrenergic antagonist for benign prostatic hyperplasia, showed signals despite relatively sparse reproductive toxicity literature. A randomized controlled trial demonstrated adverse effects on sperm parameters in healthy men (Hellstrom and Sikka, 2009), though clinical significance in real-world settings requires further investigation. For males with fertility intentions requiring these medications, clinicians should discuss potential reproductive risks and consider therapeutic alternatives or temporary discontinuation during conception attempts when medically appropriate.

This study implemented multilevel methodological strategies to enhance signal reliability and strengthen causal inference. Dechallenge analysis identified 12 drugs exhibiting symptom resolution following discontinuation, including finasteride, dutasteride, exogenous testosterone, multiple antidepressants (citalopram, escitalopram, venlafaxine), gabapentinoids, atomoxetine, the gonadotropin-releasing hormone agonist leuprorelin, minocycline, valganciclovir, verapamil, and spironolactone. The presence of dechallenge responses strengthened overall temporal plausibility between drugs and adverse events, supporting reversibility and aligning with causal assessment frameworks in pharmacoepidemiology. More critically, finasteride and etanercept simultaneously satisfied rechallenge criteria, manifesting adverse event recurrence upon drug re-administration. A positive rechallenge provides supportive evidence consistent with causality; however, within spontaneous reporting systems it remains insufficient for definitive adjudication and should be confirmed in well-designed clinical studies. Notably, the absolute numbers of dechallenge/rechallenge reports were relatively small (12 drugs for dechallenge, 2 for rechallenge) compared to widespread clinical use of these medications, reflecting the inherent challenges of documenting these phenomena in spontaneous reporting systems: patients must discontinue medication, undergo repeat fertility assessment, and have these details documented in adverse event reports—a sequence rarely completed for chronic medications. However, even small case numbers can generate statistically significant signals through disproportionality analysis when observed reporting rates substantially exceed expected background rates, as demonstrated by our requirement that all four signal detection methods simultaneously meet their respective thresholds.

The cross-database validation substantially mitigated single-source biases. FAERS and EV differ in population composition, reporting practices, and coding systems; the 19 jointly detected drugs demonstrate enhanced reliability. Finasteride, dutasteride, and testosterone showed elevated ROR values in both databases, while certain chemotherapeutic agents had higher EV reporting, potentially reflecting regional variations in chemotherapy utilization and surveillance intensity. Stratified analysis by reporter type revealed reporting bias while providing insights into risk perception: consumers focused on routine medications (alopecia treatments, testosterone, antidepressants), potentially amplified by media attention, whereas healthcare professionals concentrated on chemotherapy and immunosuppressants, reflecting systematic fertility preservation attention in oncology/autoimmune disease management.

Despite implementing multiple methodological measures, this study remains subject to inherent limitations of spontaneous reporting systems. Both FAERS and EV operate as voluntary reporting systems, characterized by underreporting and selective reporting issues. Well-known drugs may experience inflated reporting proportions due to media attention (notoriety bias), while newly marketed drugs may exhibit temporal signal fluctuation influenced by the Weber effect. The absence of exposure population denominators and precise dosage information precludes calculation of true incidence rates or assessment of dose-response relationships. Additionally, time-to-event data were documented for only 20.4% of FAERS cases (399/1,955), substantially limiting the generalizability of our temporal findings. This incomplete temporal documentation is inherent to spontaneous reporting systems where precise medication timing details are often absent. However, the observed temporal pattern (median 132 days from drug initiation to event onset) demonstrates strong biological plausibility consistent with spermatogenesis duration (≈74 days) and epididymal maturation (≈12–14 days), suggesting these may reflect phenomena, although reporting/diagnostic latency may contribute. These temporal observations should be interpreted as hypothesis-generating, requiring prospective validation.

Patients’ comorbidities (such as malignancies, immunological disorders, psychiatric conditions) may independently affect male fertility, and adjuvant treatments including radiotherapy and surgery may generate confounding effects; however, the paucity of detailed clinical information in these databases impedes effective control of these confounding factors. In addition, non-pharmacological exposures (e.g., radiofrequency exposure from mobile phone use) have been associated with impaired semen quality in prior clinical and experimental literature and could act as unmeasured confounders in spontaneous reporting data (El-Hamd and Aboeldahab, 2018). Spontaneous reporting data analysis employs disproportionality methods, evaluating whether the reporting proportion of a specific drug-event combination exceeds background levels without reference population information, utilizing metrics including ROR, PRR, and empirical Bayes geometric mean; consequently, signals merely represent abnormally elevated reporting proportions and cannot directly establish causality. Notwithstanding these limitations, FAERS and EV continue to play pivotal roles in identifying genitourinary drug adverse reactions; for example, studies investigating hematuria and drug-related osteonecrosis of the jaw have leveraged these databases to discover safety signals difficult to identify in clinical trials, thereby providing evidence for pharmaceutical safety management. The signal detection results of this study should be interpreted as safety “alerts” requiring validation through prospective cohort studies, randomized controlled trials, or real-world evidence studies.

Based on these findings, reproductive-age males using 5α-reductase inhibitors or exogenous testosterone should receive individualized fertility counseling, with fertility preservation considered for selected patients with near-term fertility goals; patients with depression requiring long-term SSRI or SNRI therapy should use shared decision-making to balance psychiatric control and reproductive priorities; young oncology patients receiving chemotherapy should strictly adhere to international guideline recommendations for pretreatment sperm cryopreservation and, when feasible, lower-reproductive-toxicity regimens. Future research should focus on conducting large-sample prospective cohort studies to elucidate true incidence rates and dose-response relationships of high-risk drugs, elucidating molecular mechanisms underlying drug effects on spermatogenesis through laboratory investigations, evaluating temporal windows and predictive factors for post-discontinuation fertility recovery, exploring interactive effects of combination drug therapies, and leveraging artificial intelligence and machine learning technologies to integrate multi-source data for establishing male infertility drug risk prediction models, thereby achieving precision reproductive health management.

## Data Availability

Publicly available datasets were analyzed in this study. FAERS data are available from the U.S. Food and Drug Administration (FDA). EudraVigilance (EV) data are publicly accessible from the European Medicines Agency (EMA). All data used were fully de-identified and analyzed in aggregated form. The main results supporting the conclusions of this article are included in the article and its Supplementary Material. Further inquiries can be directed to the corresponding author.
