# Supplementary material for: Drug-induced male infertility: a real-world study using FAERS and EudraVigilance databases
Source: Front Pharmacol. 2026 Jan 29;17:1765071. doi: 10.3389/fphar.2026.1765071 (PMC12894360; doi:10.3389/fphar.2026.1765071)
Supplement: Supplementary file 1 [file Table1.docx]

**Supplementary Table 1**

TableTwo-by-two contingency table for disproportionality analysis.

| Item | Target adverse events reported | Other adverse events reported | Total |
| --- | --- | --- | --- |
| Target drugs | a | b | a + b |
| Other drugs | c | d | c + d |
| Total | a + c | b + d | a + b + c + d |

**Supplementary Table 2**

The principles of disproportionate measurement and the criteria for signal detection.

| Method | Calculation formula | Criteria |
| --- | --- | --- |
| ROR | $ROR=\frac{a / c}{b / d}$ | a ≥ 3  95%CI (lower limit) > 1 |
|  | $SE(lnROR)=\sqrt{\frac{1}{a}+\frac{1}{b}+\frac{1}{c}+\frac{1}{d}}$ |  |
|  | $95\%CI= e^{\ln\left( ROR \right)\pm1.96se}$ |  |
| PRR | $PRR=\frac{a / (a+b)}{c / (c+d)}$ | a ≥ 3  95%CI (lower limit) > 1 |
|  | $SE(lnPRR)=\sqrt{\frac{1}{a}-\frac{1}{a+b}+\frac{1}{c}-\frac{1}{c+d}}$ |  |
|  | $95\%CI= e^{\ln\left( PRR \right)\pm1.96se}$ |  |
|  | $\chi2 =\frac{{(ad-bc)}^{2}(a+b+c+d)}{( a+b)(a+c)(c+d)(b+d)}$ | a ≥ 3  PRR ≥ 2  $\chi2\geq4$ |
| BCPNN | IC=${log}_{2}a(a+b+c+d)/(\left( a+c \right)(a+b))$ | IC025>0 |
|  | $95\%CI= E\left( \mathrm{IC} \right)\pm2V(IC)^0.5$ |  |
| MGPS | $EBGM=\frac{a(a+b+c+d)}{\left( a+c \right)(a+b)}$ | EBGM05>2 |
|  | $SE(lnEBGM)=\sqrt{\frac{1}{a}+\frac{1}{b}+\frac{1}{c}+\frac{1}{d}}$ |  |
|  | $95\%CI= e^{\ln\left( EBGM \right)\pm1.96se}$ |  |

Notes: Equation: a, number of reports containing both the target drug and target adverse drug reaction; b, number of reports containing other drugs and the target adverse drug reaction; c, number of reports of the target drug but not the adverse drug reaction of other drugs; d, number of reports of other drugs and other adverse events.

Abbreviations: ROR, reporting odds ratio; PRR, proportional reporting ratio; BCPNN, Bayesian confidence propagation neural network; EBGM, empirical Bayesian geometric mean; IC, information component; SE, standard error; 95% CI, 95% confidence interval; IC025, the lower limit of 95% CI of the IC; EBGM05, the lower limit of 95% CI of the EBGM; E(IC), the IC expectations; V(IC), the variance of IC; χ², chi-squared。

**Supplementary Table 3**

Demographic Characteristics of Target ADE Reports in the EudraVigilance Database

| Indicator | Number of Cases (%) |
| --- | --- |
| Gender |  |
| Male(%) | 1384(100.0) |
| Total(Missing) | 1384(0) |
| Age |  |
| <18(%) | 48( 3.47) |
| 18-64(%) | 756(54.62) |
| 65-85(%) | 80( 5.78) |
| ≥86(%) | 6( 0.43) |
| NotSpecified(%) | 494(35.69) |
| Total (Missing) | 1384(0) |
| Reporter |  |
| Healthcare Professional(%) | 917(66.26) |
| Non Healthcare Professional(%) | 449(32.44) |
| Not Specified(%) | 18( 1.30) |
| Total (Missing) | 1384(0) |
| Reporting Country |  |
| European Economic Area(%) | 760(54.91) |
| Non European Economic Area(%) | 624(45.09) |
| Total (Missing) | 1384(0) |
